# One Lung Wheezing due to Endobronchial Solitary Papilloma

**DOI:** 10.1155/2013/617080

**Published:** 2013-09-19

**Authors:** Suheil Artul, Faozi Artoul

**Affiliations:** ^1^Department of Radiology, Nazareth Hospital, EMMS Hospital, 16100 Nazareth, Israel; ^2^Faculty of Medicine in the Galilee, Bar-Ilan University, Safed, Israel; ^3^Meir Hospital, 44641 Kfar Saba, Israel

## Abstract

A 55-year-old man was referred to our hospital due to intolerant severe short of breathing and persistent cough for two hours. He had similar attacks in the past two years which were treated with bronchodilators. Computed tomography revealed endobronchial mass, which was endoscopically resected by laser. Histology revealed benign squamous papilloma. In this paper we will deal with the various nonspecific clinical presentations, etiopathology, predisposing factors, and diagnosing tests of these benign tumors, especially the important role of computed tomography.

## 1. Case Presentation

**Figure 1:**
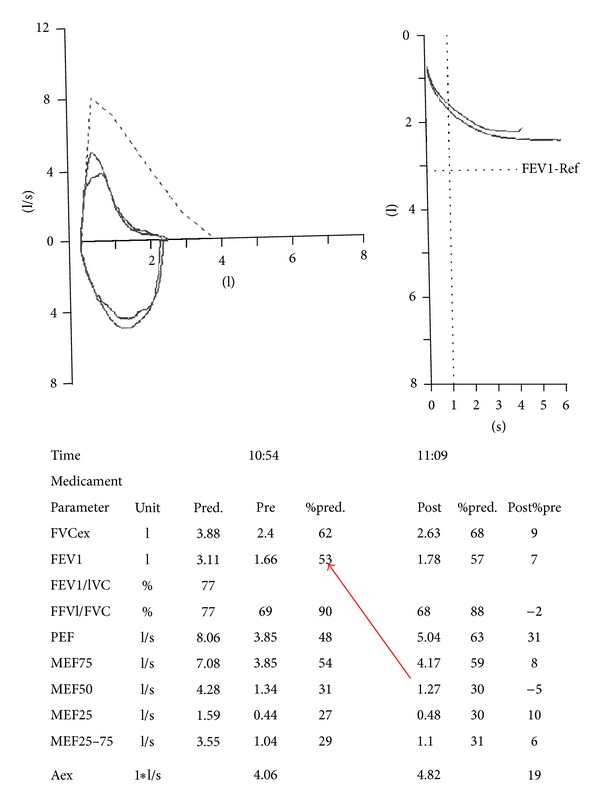
Pulmonary function test showed FEV1—53% (red arrow).

**Figure 2:**
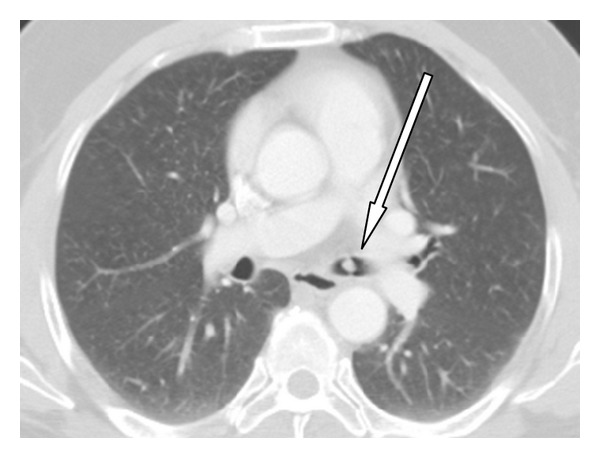
Axial CT of chest (lung window) at the level of left main pulmonary artery showing a pedunculated mass 0.5 cm in the left main bronchus (white arrow).

**Figure 3:**
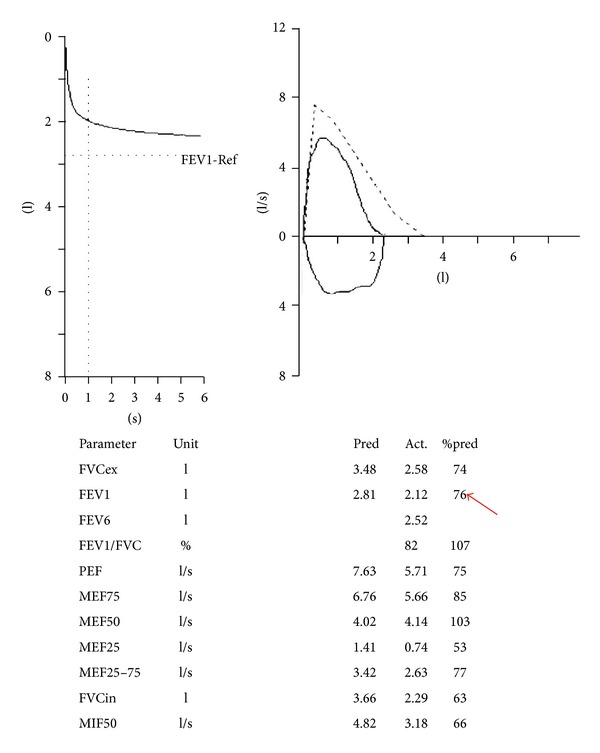
Repeated pulmonary function test after resection of the polyp resulted in improvement showing FEV1—76%.

**Figure 4:**
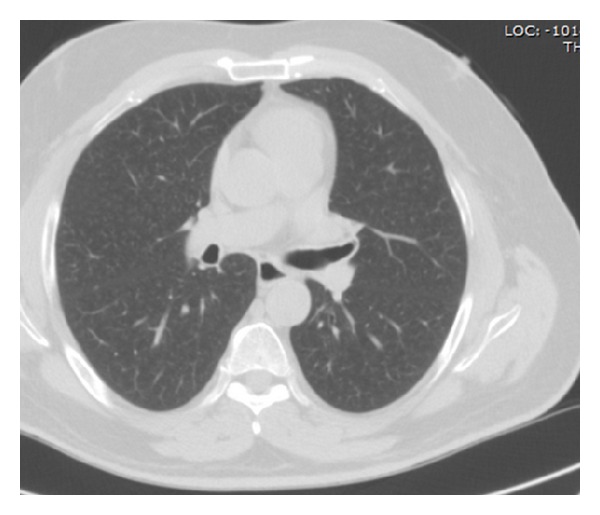
After one-year follow-up, axial noncontrast CT of chest (lung window) showing that the bronchus is still free of masses.

A 55-year-old man was referred to our hospital due to intolerant severe short of breathing and persistent cough for two hours. During the 2 years preceding presentation he suffered from less severe similar attacks, which were treated in a local clinic by bronchodilators inhalations with improvement. His past medical history revealed ischemic heart diseases and chronic obstructive airway disease. He used to smoke 30 cigarettes daily for the past 25 years. On physical examination, he had normal temperature of body 37°C, dyspnoea up to 40 breaths per minute, and, on auscultation to his lungs, perception of wheezing on the left hemithorax and normal sounds of breathing on the right one. He denied any aspiration of foreign body. Pulmonary function test (Figure 1) revealed FEV1—53%. Axial slice of chest computed tomography showed intrabronchial 0, 5 cm mass “polyp” in the left main bronchus (Figure 2 white arrow). This mass was endoscopically resected by laser without complications and histology revealed benign squamous papilloma. After the resection of the polyp, the patient did well as the symptoms regressed abruptly. Repeated pulmonary function test after resection of the polyp resulted in improvement of FEV1, which skyrocketed up to—76% (Figure 3). Because of technical reasons, HPV DNA was not tested. After one year of follow-up, the patient remains asymptomatic. Axial CT of chest (Figure 4) “lung window” showed that the bronchus still free of masses.

## 2. Discussion

Solitary endobronchial papillomas (SEP) are rare tumors. To date, less than 50 cases were described [1]. Due to low frequency, the majority of data available in the literature are case reports. Surgical resection was the gold standard for treatment, but SEP being considered as benign tumors, endobronchial treatment was successfully used (YAG Laser, electrocautery). 

 A major issue in SEP is the clinical presentation and radiological signs. The analysis of the literature shows deluding symptoms and chest X-ray presentation. The spectra of presentation vary from no symptoms to symptoms, such as haemoptysis cough, wheezing, and dypnoea. However, recurrent pneumonia and lobar collapse can occur as a secondary complication of bronchial obstruction. Our patient symptoms were dyspnoea and coughs that were diagnosed and treated as acute exacerbation of chronic obstructive pulmonary disease for the past two years.

 Radiographic features are also variable ranging from normal chest X-ray, infiltrative shadow, hilar mass, and lobar collapse. 

There is little information about the endoscopic features of SEP, as the majority of SEP which presented with radiographic abnormalities were highly suggestive of a tumoral disease and were treated surgically without previous endoscopy. Bronchoscopy was the first diagnostic tool used. In 1965, Drennan and Douglas described a solitary papilloma of the bronchus (2 × 1.5 cm) but did not find any bronchoscopic abnormalities [2]. This was surprising as the chest X-ray localized the tumor at the origin of the left lower lobe. At least, the patient experienced a coughing up of a pedunculated tumor. More recent reports found various endobronchial abnormalities. Inoue et al. described a polypoid tumor obstructing the middle lobe [3]. Katial et al. found bronchial stenosis of the left upper lobe segment, but SEP was located below the stenosis and a definite diagnosis was obtained after surgery [4].

All the lesions described by Flieder et al. were similar: polypoid, friable tan to red, glistering. and ranging in size from 0.2 to 2.5 cm. No significant differences were seen between histologic subtypes [5]. An extensive description of 8 bronchial papillomas was published more than 20 years ago [6]. Endoscopic localization was presented and confirmed the fact that SEP are located in the proximal part of the airways. SEP were described as peduncular tumors, but Barzo et al. described 5/8 tumors with a large seated base with a sharp border. They compared them to a raspberry or blackberry with a papillary structure, even to a cauliflower. All tumors were seen as tumor-like wart. Other endoscopic descriptions were available. Both retrieved white, soft, cauliflower-like tumors obstructing the bronchial lumen.

Today, the wide availability of computer tomography scan machines makes it the modality of choice to detect SEP and it is important because it can navigate the endoscopy procedure directly to the lesion.

Today bronchoscopy procedure is usually the second diagnostic modality but can be therapeutic like in our case.

Bronchial papillomas are divided morphologically into the following three main groups: (1) multiple papillomas, (2) inflammatory polyps, and (3) solitary papillomas. Solitary papillomas are the rarest type. Solitary endobronchial papillomas are divided histologically into (1) squamous, (2) glandular, or (3) mixed type papillomas.

Solitary papilloma usually presents as an endobronchial mass in the segmental bronchi and may go undetected for years. Because of the rarity of these lesions, little is known about the epidemiology and clinical behavior of solitary bronchial papillomas [7]. The relationship between human papilloma virus (HPV) and solitary papillomas has been reported. HPV proteins E6 and E7 can bind the protein products of tumor suppressor gene p53 and the retinoblastoma protein and can induce cellular proliferation and dysplasia. Different HPV subtypes show different binding properties. HPV types 6 and 11 are considered to be the cause of papillomas [4]. Endobronchial foreign bodies are reported as of another etiologic origin [7]. There seems to be a very close relationship between this type of papilloma and smoking habits. Passive smoking also needs to be taken into account in female cases [8]. Our case was also a heavy smoker. The age distribution of the cases is in a very wide range of 22 to 80 years (mean, 58.3 years) and the highest incidence of solitary bronchial papilloma is seen in the 6th decade, and our patient was rather 55 years old. These tumors are six times more common in men than in women [9]. Most tumors were located in the segmental or more central bronchi (86%), while the remainders were in subsegmental or peripheral bronchi. The papilloma usually spreads exophytically; however rarely, it penetrates the deeper layers of the bronchial wall (“ice-mountain” type), that is why these tumors can be resected safely endoscopically with low rate of recurrence [9]. 

In this paper we reported a case of SEP which was presented clinically with dyspnoea and one lung wheezing and was treated successfully with laser.

## 3. Conclusion and Learning Points

SEP is a rare disease and could be caused by smoking. Computed tomography plays a crucial role in diagnosing patients with unilateral lung wheezing, prolonged cough, and dyspnoea. Endoscopic resection by laser is the treatment of choice for noncomplicated SEP.
